# Development of Environmentally Friendly Atom Transfer Radical Polymerization

**DOI:** 10.3390/polym12091987

**Published:** 2020-08-31

**Authors:** Ming Yuan, Xuetao Cui, Wenxian Zhu, Huadong Tang

**Affiliations:** Institute of Industrial Catalysis, College of Chemical Engineering, Zhejiang University of Technology, Hangzhou 310014, Zhejiang, China; 13750872796@163.com (M.Y.); cxt17816038659@163.com (X.C.); zwx11176680@163.com (W.Z.)

**Keywords:** ATRP, catalyst, environmental friendliness, iron complex, enzyme, metal-free catalyst

## Abstract

Atom transfer radical polymerization (ATRP) is one of the most successful techniques for the preparation of well-defined polymers with controllable molecular weights, narrow molecular weight distributions, specific macromolecular architectures, and precisely designed functionalities. ATRP usually involves transition-metal complex as catalyst. As the most commonly used copper complex catalyst is usually biologically toxic and environmentally unsafe, considerable interest has been focused on iron complex, enzyme, and metal-free catalysts owing to their low toxicity, inexpensive cost, commercial availability and environmental friendliness. This review aims to provide a comprehensive understanding of iron catalyst used in normal, reverse, AGET, ICAR, GAMA, and SARA ATRP, enzyme as well as metal-free catalyst mediated ATRP in the point of view of catalytic activity, initiation efficiency, and polymerization controllability. The principle of ATRP and the development of iron ligand are briefly discussed. The recent development of enzyme-mediated ATRP, the latest research progress on metal-free ATRP, and the application of metal-free ATRP in interdisciplinary areas are highlighted in sections. The prospects and challenges of these three ATRP techniques are also described in the review.

## 1. Introduction

Since it was discovered independently by Matyjaszewski and by Sawamoto in 1995 [1,2], atom transfer radical polymerization (ATRP) has become one of the most powerful tools for the preparation of well-defined polymers with controlled molecular weights, narrow molecular weight distributions, and designable molecular architectures. Typically, the ATRP system is composed of monomer, initiator and catalyst. The most important component is the catalyst, which is a key factor to the ‘living’/controlled characteristics of polymerization. Up to now, a large variety of transition metal complexes—such as Cu, Ru, Mo, Rh, Ni, Pd, Co coordination compounds—have been successfully employed as catalysts in ATRP systems [3,4,5,6,7,8,9,10,11,12,13,14,15,16,17,18,19,20,21,22,23,24].

The environment-friendly aspects of ATRP have attracted a lot of interest in recent years. In an ideal situation, a good ATRP catalyst could realize: (1) synthesizing polymer with desired molecular weight and narrow polydispersity; (2) high catalytic activity for the polymerization reaction; (3) a little amount of residual catalyst in the polymer. To the best of our knowledge, the fulfillment of these requirements still remains a challenge. Since the ATRP of methyl methacrylate and styrene with iron complex as catalyst was first reported in 1997 [25,26], iron coordination compounds have been one of the most promising environmentally friendly catalysts because of their low toxicity, inexpensive cost, and abundant commercial availability [27]. Besides iron complex catalyst, enzyme-mediated ATRP has also attracted considerable interest due to its high efficiency and selectivity, mild reaction conditions, and excellent biocompatibility [28]. Recently, metal-free ATRP has emerged as a green and sustainable strategy for precise polymer synthesis [29].

Matyjaszewski and coworkers have summarized iron-catalyzed ATRP recently on the basis of the structures and properties of iron ligands, the effects of ligands on the polymerization rate, and the development of initiating systems for activator regeneration [30]. Xie et al. also reviewed iron-catalyzed ATRP based on mechanistic considerations and the types of iron complex and iron ligand [31]. Hawker and de Alaniz presented a review on metal-free ATRP and discussed its catalysis principle, catalyst structure, monomer scope, and application in the synthesis of architecturally complex materials [29]. As enzymes could be denatured and deactivated by heavy metal catalyst, there are only a few reports on enzyme-mediated ATRP [28].

Herein, this review aims to provide a comprehensive understanding of these three types of environmentally friendly ATRP technology and primarily focused on the catalytic activity, initiation efficiency, polymerization controllability, and environmental friendliness. The latest research progress on iron complex-catalyzed ATRP of a large variety of monomers, the recent developments of enzyme-mediated ATRP, and the application of metal-free catalyst mediated ATRP in interdisciplinary areas are highlighted in sections. The prospects and challenges of these three ATRP techniques are also described in the review.

## 2. Applications of Iron Catalyst in Various ATRP Systems

### 2.1. Applications of Iron Catalyst in Normal ATRP

**Figure 1:**
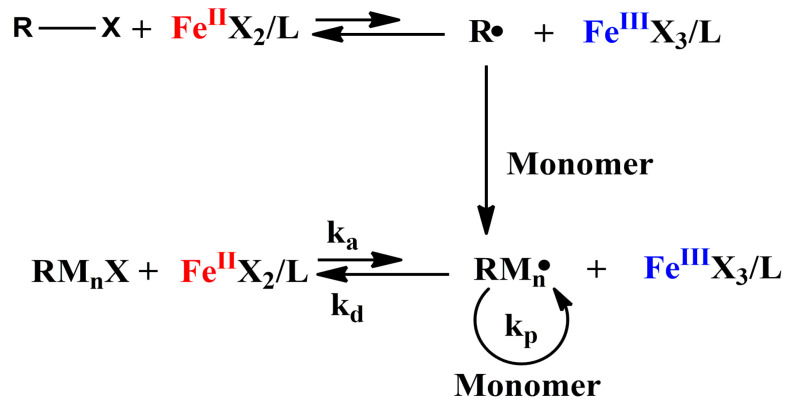
Mechanism of normal ATRP catalyzed by iron(II) complex.

Transition metal complex and organic halide are generally used as catalyst and initiator respectively in early ATRP system. The earliest catalyst in a normal ATRP was a copper complex (CuCl/Bipyridine) [1], which had excellent catalytic activity and good control for the polymerization of multiple monomers. Compared to copper complex, iron complex has broader application prospects because of its environmentally friendly nature. The mechanism of normal ATRP catalyzed by iron complex is shown in Figure 1.

Matyjaszewski group first reported in 1997 that iron complex-catalyzed normal ATRP of styrene (St) and methyl methacrylate (MMA) with (1-bromoethyl)benzene (PEBr) as an initiator [25]. They investigated the effect of different ligands such as tri-n-butylphosphine, tri-n-butylamine, and triphenylphosphine on the polymerization and found that the polymerization catalyzed by FeBr_2_/tri-n-butylamine had well-controlled characteristics for styrene and methyl methacrylate, but not for acrylate. Sawamoto et al. also reported an iron complex-catalyzed normal ATRP of MMA in 1997 using iron(II) bis(triphenylphosphine)dichloride [FeCl_2_(PPh_3_)_2_] as catalyst [26]. The number average molecular weight of obtained poly(methyl methacrylate) (PMMA) increased linearly with the increase of monomer conversion, and the molecular weight distribution was narrow (*M*_w_/*M*_n_ = 1.1–1.3). Later, iron complexes with halide anions as ligands were found to catalyze controlled polymerizations of acrylates by Matyjaszewski and coworkers in 2000 [32]. The residual catalysts could be readily removed from the polymer products because of their ionic nature. However, this polymerization system was not applicable for ATRP of styrene probably due to the involvement of cationic polymerization.

Sawamoto et al. used iron complex [Fe(Cp)I(CO)_2_; Cp = cyclopentadienyl] as catalyst in normal ATRP of acrylates, finding that the synthesized polymers had controlled molecular weights and narrow molecular weight distributions (*M*_w_/*M*_n_ < 1.2) [33]. This catalytic system was also applicable for the synthesis of poly(methyl acrylate)-*b*-poly(styrene) and poly(butyl acrylate)-*b*-poly(styrene) block copolymers, but the controllability of the polymerization was decreased when [(CH_3_)_2_C(CO_2_CH_3_)CH_2_C(CH_3_)(CO_2_CH_3_)Br] or [(CH_3_)_2_C(CO_2_CH_3_)CH_2_C(CH_3_)(CO_2_CH_3_)Cl] was used as an initiator. Sawamoto and coworkers later found that the iron bromide complexed with butylphosphine had an excellent activity/controllability in the normal ATRP of MMA [34]. The polymerization reached over 90% conversion in 5 h, producing PMMA with narrow molecular weight distributions (*M*_w_/*M*_n_ = 1.20–1.32). Yan and coworkers discovered that the polymerization of styrene was controlled when using low toxic organic acid as ligand in iron mediated ATRP, but the obtained polystyrene (PS) had a relatively broad molecular weight distribution (*M*_w_/*M*_n_ = 1.50) [35].

Gibson et al. demonstrated that a four-coordinated iron(II) complex bearing α-diimine ligands had high catalytic activity in normal ATRP of styrene [36]. A five-coordinated iron(II) complex containing tridentate nitrogen donor ligands was also found very effective for styrene [37]. They investigated the effects of different structures of catalyst on the reaction rate of ATRP and revealed that α-diimine ligands bearing electron-donating groups increased the polymerization rate but the ligands bearing electron-withdrawing substituents decreased the polymerization rate. Zhang and coworkers reported that iron(II) ligated with *N*-(n-hexyl)-2-pyridylmethanimine (NHPMI) was an effective catalyst for ATRP of MMA [38]. The molar ratio of iron/NHPMI had relatively large impacts on the controllability of the polymerization. The molecular weight distribution of produced PMMA ranged from 1.25 to 2.50. Wang et al. proved that iron(II) coordinated with tris(3,6-dioxaheptyl)amine (TDA) was an excellent catalyst in normal ATRP of styrene with (1-chloroethyl)benzene (PECl) or PEBr as an initiator [39]. The residual catalyst could be easily removed out of the polymer product because of the good water solubility of TDA.

Xue et al. reported that using 2-[(diphenylphosphino)-methyl]pyridine (DPPMP) as ligand in an iron mediated normal ATRP of MMA had well-controlled characteristics [40]. The molecular weight of PMMA increased linearly with the increase of monomer conversion. They investigated the effect of different solvents (e.g., p-xylene, toluene, and anisole) on the polymerization, and found that the controllability was decreased when using p-xylene or anisole as a solvent and only PMMA prepared in toluene had low polydispersities (*M*_w_/*M*_n_ = 1.1–1.3). The system was not applicable for controlled polymerizations of methyl acrylate (MA) and butyl acrylate (BA). Recently, Gao et al. claimed that the iron(II) complexed with anilidoimine ligands showed excellent catalytic performances in ATRP of MMA [41]. Nagashima and coworkers described that a trinuclear iron(II) complex with 1,4,7-trimethyl-1,4,7-triazacyclononane (Me_3_TACN) as ligand was an effective catalyst for normal ATRP of styrene [42]. The residual catalyst in the polystyrene could be removed by a simple washing process. Nagashima et al. later also reported that the [(i-Pr)_3_TACN]FeX_2_ complex (TACN = *N*,*N*,*N*-substituted-1,4,9-triazanonane) had a high catalytic activity in ATRP of styrene and MMA [43]. They further found that the iron complex [{(cyclopentyl)_3_TACN}FeBr_2_]_n_ could catalyze well-controlled polymerizations of styrene, BA, and MMA [44]. The PS-*b*-PMMA block polymer had been synthesized using this catalyst at a low catalyst concentration of 59 ppm.

### 2.2. Applications of Iron Catalyst in Reverse ATRP

**Figure 2:**
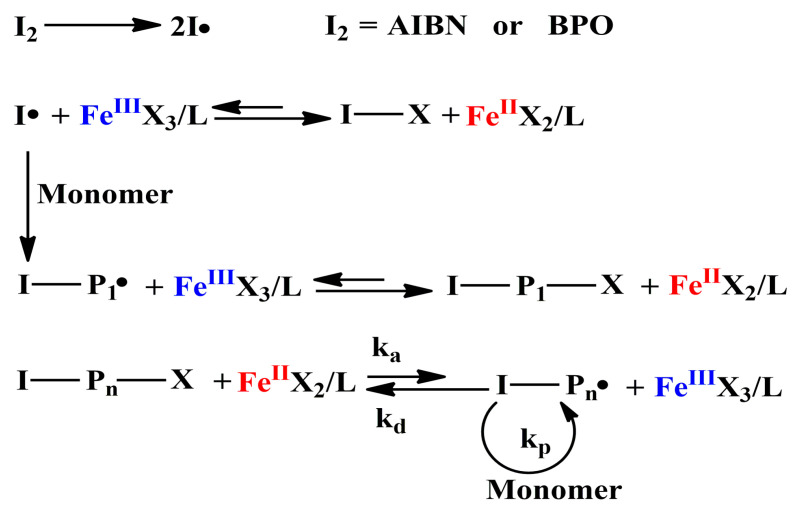
Mechanism of reverse ATRP catalyzed by iron(III) catalyst.

Normal ATRP usually presents some limitations such as requirement of relatively large amount of catalyst and instability of the lower oxidation state of transition metal complex. The use of oxidatively stable catalyst in ATRP is a favorable approach to solve the problem. Reverse ATRP catalyzed by air-stable higher oxidation state metal complex has been successfully applied to prepare well-designed polymers. The mechanism of iron mediated reverse ATRP is shown in Figure 2.

The iron mediated reverse ATRP of MMA with triphenylphosphine as ligand and azodiisobutyronitrile (AIBN) as initiator was first reported by Teyssié and coworkers in 1998 [45]. From then on, a series of iron complex mediated reverse ATRP of vinyl monomers had been investigated. Qiu et al. prepared well-defined PMMA with high molecular weight (*M*_n_ = 171,800 Da) and narrow molecular weight distribution (*M*_w_/*M*_n_ = 1.13) by a reverse ATRP with 1,1,2,2-tetraphenyl-1,2-ethanediol (TPED)/FeCl_3_/PPh_3_ as catalyst [46]. They later investigated the polymerization of MMA by using diethyl 2,3-dicyano-2,3-diphenylsuccinate (DCDPS)/FeCl_3_/PPh_3_ as a catalyst [47]. The polymerization could be well-controlled even at high monomer conversions.

Zhu and coworkers conducted a reverse ATRP of MMA using iron(III)/pyromellitic acid as a catalyst and AIBN as an initiator [48]. However, they found that this catalytic system was only effective for methacrylates but not for acrylates. Zhu et al. later reported an iron mediated reverse ATRP of MMA using 2,2-azobis(2-methylpropionamidine) dihydrochloride (V-50) as initiator and *N*,*N*-butyldithiocarbamate ferrum (Fe(S_2_CN(C_4_H_9_)_2_)_3_) as catalyst, but the monomer conversion and polymerization rate in this system were relatively low [49]. Ferro and coworkers showed that the Fe(BOX)Cl_3_ (BOX = 1,1-bis(4,4-dimethyl-1,3-oxazolin-2-yl)ethane) was an excellent catalyst for the reverse ATRP of styrene [50]. Ferro et al. investigated the reverse ATRP of styrene initiated by TPED and catalyzed by three iron(III) complexes—namely Fe^III^ coordinated with BOX, 3,5-dimethyl-bispyrazolylmethane as well as 2,2′-dipyridyl—and found that only TPED/FeCl_3_/BOX produced polystyrene with controlled molecular weights and narrow molecular weight distributions [51].

Shaver and coworkers revealed that reverse ATRP of MMA and styrene could be achieved using α-diimine iron complexes as catalysts and AIBN as initiator [52]. Based on the advantages of their iron complex, a series of monomers such as MA [32,52], stearyl methacrylate (SMA) [53], n-hexadecyl methacrylate (HMA) [54], 2-hydroxyethyl methacrylate (HEMA) [55], acrylonitrile (AN) [56], n-docosyl acrylate (DA) [57], and methacrylonitrile (MAN) had been polymerized by reverse ATRP [58].

### 2.3. Applications of Iron Catalyst in Initiators for Continuous Activator Regeneration (ICAR) ATRP

**Figure 3:**
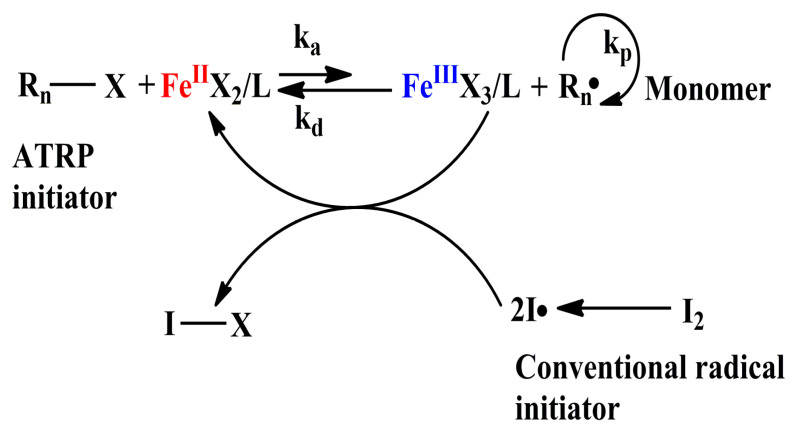
Mechanism of ICAR ATRP catalyzed by iron(III) catalyst.

A relatively large amount of catalyst is required in normal ATRP and reverse ATRP system. Consequently, a relatively high concentration of catalyst residue is inevitably left in the polymer and brings difficulty in the purification of polymer product after polymerization. In order to overcome this limitation, the initiators for continuous activator regeneration atom transfer radical polymerization (ICAR ATRP) had been developed [59,60]. Iron catalyst has been successfully applied to ICAR ATRP. The mechanism of iron mediated ICAR ATRP is shown in Figure 3.

In an iron-catalyzed ICAR ATRP system, radicals are generated by a conventional radical initiator such as AIBN. The polymerization possesses highly controllable characteristics even at very low concentration of catalyst. Zhu and coworkers firstly reported an iron mediated ICAR ATRP of MMA and styrene in 2010 [60]. They investigated the effects of different polymerization conditions on the ICAR ATRP, finding that the ICAR ATRP of styrene could be achieved even if the concentration of iron(III) catalyst was as low as 50 ppm. However, this system showed insufficient catalytical activity for the polymerization of MMA because of the intrinsic low activity of iron catalyst for polar monomers. Zhu et al. later demonstrated a controlled ICAR ATRP of MMA using iron(III) complexed with PPh_3_ as a catalyst, bifunctional 1,4-(2-bromo-2-methylpropionato)benzene (BMPB_2_) as an initiator, and AIBN as a thermal radical initiator [61]. This catalytic system showed excellent activities and promoted the polymerization of MMA even at a very low catalyst concentration of 30 ppm.

Wang and coworkers performed an ICAR ATRP of MMA using ethyl 2-bromoisobutyrate (EBiB) as an initiator, AIBN as a thermal radical initiator, and FeCl_3_·6H_2_O/succinic acid as a catalyst [62]. The polymerization could produce PMMA with narrow molecular weight distribution (*M*_w_/*M*_n_ = 1.20–1.50) at low catalyst concentrations (30–100 ppm). However, a relatively slower polymerization rate was observed in this system, and the polymerization controllability was found to decrease with the increase of AIBN, indicating the presence of coupling side reactions of macromolecular radicals in the polymerization.

Matyjaszewski et al. reported an iron(III) complex mediated ICAR ATRP of MMA using AIBN as thermal initiator at a catalyst concentration of 100 ppm [63]. Later, an iron(III) complex mediated ICAR ATRP of styrene using 1,1-azobis(cyclohexanecarbonitrile) (ACHN) as thermal initiator had also been reported by his group [64]. The obtained polystyrene had a narrow molecular weight distribution with a polydispersity index (PDI, PDI = *M*_w_/*M*_n_) of 1.29. The polymerization rate was found to be largely depended on the amount of ACHN. Matyjaszewski and coworkers investigated ICAR ATRP of MMA and styrene catalyzed by iron-based *N*-heterocyclic carbene (FeX_3_(NHC)) complexes, finding that both FeX_3_(IDipp) (IDipp = 1,3-bis(2,6-diisopropyl-phenyl)-imidazol- 2-ylidene) and FeX_3_(HIDipp) (HIDipp = 1,3-bis(2,6-diisopropyl-phenyl)imidazolidin-2-ylidene) had excellent catalytical activities and produced PMMA and PS with controlled molecular weights and narrow molecular weight distributions (*M*_w_/*M*_n_ = 1.15–1.40) [65].

### 2.4. Applications of Iron Catalyst in Activators Generated by Electron Transfer (AGET) ATRP

**Figure 4:**
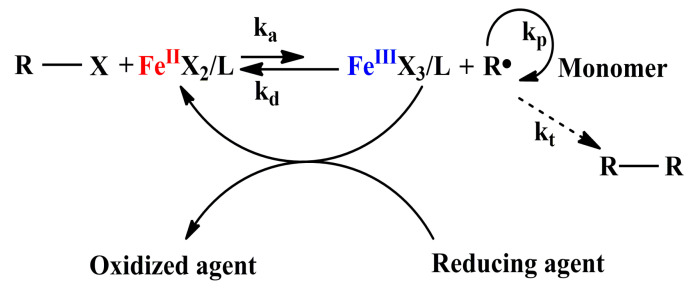
Mechanism of AGET ATRP catalyzed by iron(III) catalyst.

Activators generated by electron transfer (AGET) ATRP was first developed by Matyjaszewski [66]. Compared to normal ATRP, AGET ATRP has a better chance for industrial applications owing to its lower requirement of catalyst. In a typical AGET ATRP, alkyl halide and transition metal complex in its oxidatively stable state are used as initiator and catalyst respectively. The activators are produced by in situ reduction of the oxidatively stable metal complex with tin(II) 2-ethylhexanoate [Sn(EH)_2_] [67], ascorbic acid (VC) [68,69], or other reducing agents [70]. Iron catalysts are highly promising in AGET ATRP system because of their environmental friendliness, naturally abundant features, and biocompatibilities. A descriptive mechanism of AGET ATRP catalyzed by iron complex is shown in Figure 4.

The AGET ATRP of MMA using FeCl_3_ complexed with iminodiacetic acid (IDA) as catalyst and VC as reducing agent had been reported by Zhang and his colleagues in 2008 [68]. The polymerization was well-controlled even in the presence of a limited amount of air, producing PMMA with narrow molecular weight distributions (*M*_w_/*M*_n_ = 1.31–1.44). Zhang et al. used FeCl_3_/PPh_3_ complex as a catalyst and VC as a reducing agent in AGET ATRP of MMA [71]. However, the above mentioned two catalytic systems showed relatively low catalytic performance due to low initiator efficiency. Zhang and coworkers later reported an iron(III) mediated AGET ATRP of styrene using TDA as a ligand and 1,3,5-(2′-bromo-2′-methylpropionato)benzene (BMPB) as an initiator [72].

The iron-based AGET ATRP of styrene derivatives including 4-methylstyrene (MS), 4-acetoxystyrene (AS), and 4-tert-butylstyrene (tBS) have been explored by Sen and his coworkers using PEBr as an initiator, FeBr_3_/tributylamine as a catalyst, and Sn(EH)_2_ as a reducing agent [73]. Well-defined PS-*b*-PMMA block copolymer had been prepared using the catalytic system. The monomer conversion was revealed to be determined by the activity of the reducing agent and the yield would be dramatically decreased if a weak reducing agent was used in the polymerization. As the requirement of a large amount of reducing agent actually limits the industrial application of iron(III) mediated AGET ATRP, it is of great importance to decrease the amount of reducing agent and find low-cost and commercially available reducing agent. Xue and coworkers recently found that the iron(III) mediated ATRP of MMA could be successfully achieved using trimethylphosphite (TMP) and tributylphosphine (TBP) as ligands even in the absence of reducing agent [74].

Zhu and his colleagues reported an iron mediated AGET ATRP of styrene in the presence of Fe(OH)_3_ using commercially available tetra-n-butylphosphonium bromide (TBPBr) or tetrabutylammonium bromide (TBABr) as the ligands [75]. They claimed that the polymerization could occur at the catalyst concentration as low as ppm level. However, as the inorganic base Fe(OH)_3_ was practically insoluble in most organic solvents, it was difficult to calculate the exact amount of the iron catalyst participated in the reaction. Yan and coworkers used 1-butyl-3-methyl imidazolium hydroxide as an additive to enhance the iron mediated AGET ATRP of MMA [76]. Compared to Fe(OH)_3_, 1-butyl-3-methyl imidazolium hydroxide was readily soluble in MMA and other organic solvents. Therefore, it was feasible to quantify the amount of 1-butyl-3-methyl imidazolium hydroxide. They investigated the effect of the molar ratio of 1-butyl-3-methyl imidazolium hydroxide to iron complex on the polymerization, finding that the polymerization was controlled only in appropriate ratios of 10.8:1–15.6:1. Yan et al. later studied the effects of the other imidazolium-type ionic liquids (ILs) such as 1-butyl-3-methyl imidazolium carbonate ([Bmim][CO_3_]), 1-butyl-3-methyl imidazolium phosphate ([Bmim][PO_4_]) and 1-butyl-3-methyl imidazolium bicarbonate ([Bmim][HCO_3_]) on the iron mediated AGET ATRP of MMA [77].

Cellesi and coworkers discovered that the iron(III) complexed with commercial porphyrin ligand was an excellent catalyst for the AGET ATRP of poly(ethylene glycol) methyl ether methacrylate (PEGMA) [78]. Recently, Bai et al. found that iron(III) ligated with 1-butyl-3-methylimidazolium hexafluorophosphate (BMIMPF_6_) was an excellent catalyst for the AGET ATRP of MMA [79]. Fe(0) wire was used as a reducing agent in this polymerization and it could be recycled and reused.

### 2.5. Applications of Iron Catalyst in Generation of Activators by Monomer Addition (GAMA) ATRP

**Figure 5:**
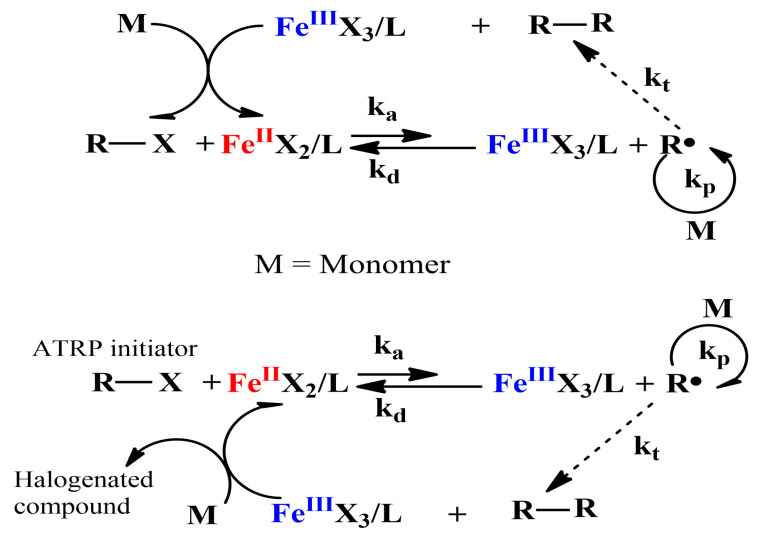
Mechanism of GAMA ATRP catalyzed by iron (III) catalyst.

The GAMA ATRP is usually conducted without the use of conventional free radical initiators or reducing agent. The Fe(II) complex is generated by the reaction of Fe(III) complex and monomer due to the oxidizing power of FeX_3_. The mechanism of GAMA ATRP catalyzed by iron complex is shown in Figure 5.

Noh and coworkers reported an iron(III) mediated ATRP of MMA using phosphorus as ligand in the absence of a conventional free radical initiator or reducing agent [80]. The effects of iron complex and ligand on the polymerization have been investigated, finding that the FeBr_3_/DPPP/EBiB [DPPP = 2-(diphenylphosphino)pyridine] system showed the highest controllability and produced well-defined PMMA (*M*_n_ = 1.75 × 10^4^ Da, PDI = 1.18). In addition, controlled polymerizations of butyl methacrylate, methyl acrylate, and styrene were also achieved by using FeBr_3_/DPPP as a catalyst. For comparison, a normal ATRP of MMA using FeBr_2_/phosphorus as the catalyst was also investigated. However, the molecular weights of produced PMMA were higher than theoretical values, and a relatively higher molecular weight distribution (PDI = 1.37) was obtained. These results indicated that the FeBr_3_/phosphorus showed a better controllability than the FeBr_2_/phosphorus for the polymerization of MMA. Later, a series of Fe(III)/phosphorus mediated GAMA ATRP have been reported by their group, and a large number of well-defined polymers and block copolymers have been synthesized [74,81,82,83].

Kamigaito and coworkers conducted a FeCl_3_/TnBP [TnBP = tri(n-butyl)phosphine] mediated polymerization of styrene without the use of conventional radical initiator and reducing agent [84]. The polymerization was well-controlled and yielded poly(styrene) (PS) with a low polydispersity index (PDI = 1.19). Moreover, this polymerization was also successfully applied to the copolymerization of styrene with other monomers including MA, MMA, and BA. Later, the FeX_3_/nitrogen ligand (X = Cl, Br) catalyzed polymerizations of styrene, MMA and MA in the absence of conventional radical initiator and reducing agent were also achieved in their group [85]. Well-defined PS (*M*_n_ = 1.03 × 10^4^ Da, PDI = 1.11), PMMA (*M*_n_ = 9.70×10^3^ Da, PDI = 1.26), PMA (*M*_n_ = 8.50 × 10^3^ Da, PDI = 1.15), and PMMA-*b*-PS (*M*_n_ = 2.01 × 10^4^ Da, PDI = 1.31) block copolymer were successfully prepared. A mechanism investigation revealed that FeCl_3_ was converted into [Fe(III)Cl_4_^‒^] and [Fe(III)Cl_2_^+^] in the presence of phosphine or nitrogen ligand. The [Fe(III)Cl_2_^+^] interacted with phosphorus ligand give Fe(III)Cl_2_(PR_3_)^+^ or [Fe(II)Cl_2_(PR_3_)^•+^] species, which were the active catalysts for ATRP. Matyjaszewski and coworkers reported that in the Fe(III)X_3_/phosphorus mediated ATRP of styrene [86]. The phosphines could directly reduce Fe(III) to Fe(II) and could also act as a ligand coordinated with iron to form efficient ATRP catalyst.

Recently, Xue et al. reported a FeBr_3_/TPP (TPP = triphenylphosphine) catalyzed polymerization of MMA in the absence of conventional free radical initiators or reducing agent. The system could also be applied to the polymerization of butyl methacrylate (BMA) and styrene (St) [87]. The mechanism of the polymerization has been investigated, and it was found that the polymerization was initiated by Ph_3_PBr_2_ or Ph_3_PBr_4_ generated from the reaction between TPP and FeBr_3_.

### 2.6. Applications of Iron Catalyst in Supplemental Activator and Reducing Agent (SARA) ATRP

**Figure 6:**
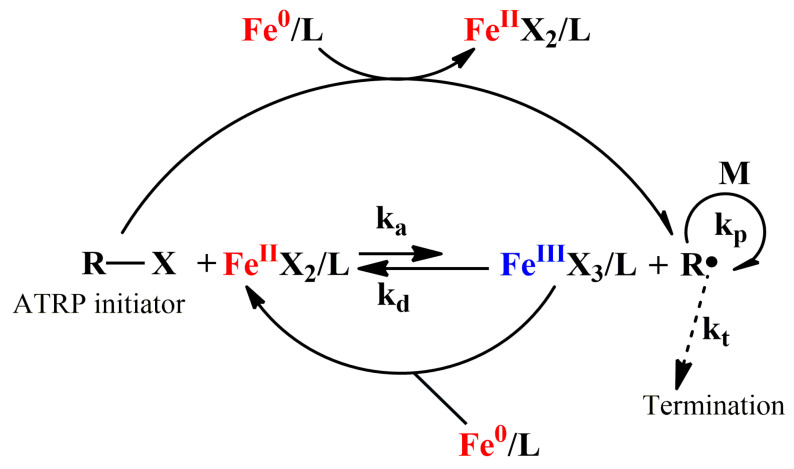
Mechanism of SARA ATRP catalyzed Fe(0) catalyst.

Fe(0) is a kind of reducing agents in iron mediated AGET ATRP. It can also act as a supplemental activator and react with ATRP initiator to induce a polymerization [63,88]. This system is termed as supplemental activator and reducing agent ATRP (SARA ATRP). The mechanism of SARA ATRP is as shown in Figure 6.

Coelho and coworkers reported a Fe(0)/Cu(II) based SARA ATRP of 2-(dimethylamino)ethyl methacrylate (DMAEMA) [89]. The molecular weights of obtained poly(DMAEMA) (PDMAEMA) increased linearly with the increase of monomer conversions and the molecular weight distribution was narrow (PDI = 1.13). Moreover, the polymerization of DMAEMA was also realized by using bromo-telechelic mPEG (mPEG-Br) or cholesteryl-2-bromoisobutyrate (CHO-Br) as a macroinitiator, producing corresponding PEG-*b*-PDMAEMA and CHO-*b*-PDMAEMA block copolymers. They later carried out the Fe(0)/Cu(II) based SARA ATRP of MA and glycidyl methacrylate (GMA), and prepared PMA and poly(GMA) with low polydispersity index (PDI = 1.08 and 1.27 respectively) [90].

### 2.7. Developments of Iron Ligands in ATRP

The ligand is a key factor in iron mediated ATRP. The redox potential of the metal core is controlled by the ligand around the center iron ions. Therefore, the polymerization rate and the controllability are practically dependent on the ligands. A large variety of phosphorous, nitrogen, and oxygen compounds have been used as iron ligands in ATRP, and these ligands are primarily classified as nitrogen-based ligand, phosphorous ligand, organic acid-based ligand, and onium salt-based ligand.

#### 2.7.1. Nitrogen-Based Ligands

As nitrogen ligand has shown excellent performances in copper mediated ATRP, it has also been largely used in iron mediated polymerizations. The first nitrogen ligand used in iron complex catalyzed ATRP was tri-n-butylamine (TnBA), reported by Matyjaszewski in 1997 [25]. The FeBr_2_/TnBA complex showed very high activity and catalyzed well-controlled polymerization of styrene and methyl methacrylate. Other typical nitrogen ligands include dibutylamine (DnBA) [85], n-butylamine (nBA) [85], TDA [91], tetramethylethylenediamine (TMEDA) [92,93], pentamethyldiethylenetriamine (PMDETA) [94,95], *N*,*N*,*N*′,*N*″,*N*″-penta(methyl acylate)diethylene- triamine (MA_5_-DETA) [96], 2,2′-bipyridine (bpy) [97], 4,4′-dinonyl-2,2′-dipyridyl (dNbpy) [98], tris(2-dimethylaminoethyl)amine (Me_6_TREN) [88], NHPMI, and 1,3-bis-(dimethylamino)propane (TPDA) [38,99].

Sawamoto and coworkers reported an iron mediated ATRP of MMA with a chiral compound, (*R*)-*N*,*N*-dimethyl-1-(2-(diphenylphosphino)phenyl)-ethanamine, as ligand. The polymerization could reach 92% conversion in 23 h [99]. Matyjaszewski group showed an iron mediated polymerization of vinyl acetate (VAc) using PMDETA as ligand [100]. They found that the iron complex in this system actually acted as a redox initiator but not a catalyst. Interestingly, the synthesized poly(vinyl acetate) (PVAc) was an excellent macroinitiator for ATRP of styrene and BA. They later reported a Fe(0) mediated ATRP of MA with Me_6_TREN as ligand [88]. The polymerization was well controlled but only reached a low conversion of 16% due to the formation of inefficient deactivators.

Zhang and coworkers presented an iron mediated ATRP of MMA using MA_5_-DETA as a ligand [101]. The catalyst promoted well-controlled ATRP of MMA with high initiation efficiency. Wu et al. conducted an iron mediated ATRP of MMA using hexamethylene tetramine (HMTA) as ligand, and prepared PMMA with controlled molecular weights and narrow molecular weight distributions (*M*_w_/*M*_n_ = 1.24–1.41) [102]. Baruah et al. performed ATRP of HMA using tris(2,2′-bipyridine)iron(III) complex as catalyst and CBr_4_ as initiator [54]. The effects of monomer, initiator, and catalyst concentration on the controllability of the polymerization were investigated. ATRP of styrene with FeBr_2_/Fe(0)/dNbpy as a catalyst had been reported and the polymerization produced polystyrene with narrow molecular weight distributions (*M*_w_/*M*_n_ = 1.17–1.27) [103]. The iron mediated ATRP of MMA with [*N*,*N*′-diphenyl-*N*,*N*′-di(quinoline-2-methyl)]-1,2-ethylene diamine (DPDQMEDA), (1*R*,2*R*)-(–)-*N*,*N*′-di(quinoline-2-methyl)di-iminocyclohexane (DQMDICH) and [*N*,*N*′-dioctyl-*N*,*N*′-di(quinoline-2-methyl)]-1,2-ethylene (DODQMEDA) as ligands had also been reported [104]. Except for the nitrogen-based ligands mentioned above, the tridentate diiminopyridine (DOIEP) and diaminopyridine (DOAEP) were also used in iron mediated ATRP [105].

#### 2.7.2. Phosphorous Ligands

Phosphorous ligands exhibit excellent performances in iron mediated ATRP. The first phosphorous ligand used in iron mediated ATRP was TPP, reported by Sawamoto in 1997 [26]. A large number of phosphorus ligands such as TnBP [25], TMP [74], triethyl phosphite (TEP) [82], TPP [106], tricyclohexyl phosphine (TCHP) [107], tris(4-methoxyphenyl)phosphine (TMPP) [86], trichlorophenyl phosphine (TCPP) [107], tris(2,4,6-trimethoxyphenyl)phosphine (TTMPP) [86], DPPP [108], DPPMP [40], 2-(diphenylphosphino) benzaldehyde (DPPB) [80], diphenyl-(2-methoxyphenyl)phosphine (DPMPP) [83], *N*-(2-diphenyl-phosphinobenzylidene)- aniline (DPPBA) [80], *N*,*N*-dimethyl-(2-(diphenylphosphino)phenyl)-methanamine (DMDPM) [99], (*R*)-*N*,*N*-dimethyl-1-(2-(diphenylphosphino)-phenyl)ethanamine (DMDPE) [99], *N*,*N*-dimethyl- (2-(dicyclohexylphosphino)-phenyl)methanamine (DMDCM) [99], bis(diphenylphosphino)- methane (BDPPM) [74], and 1,2-bis(diphenylphosphino)ethane (BDPPE) have been developed for iron mediated ATRP [74].

Sawamoto group reported an iron mediated ATRP of PEGMA using TPP as a ligand and prepared corresponding polymers with controlled molecular weights and narrow molecular weight distributions (PDI < 1.20) [107]. They investigated the effect of different ligands such as TPP, TMPP, and TCPP on the polymerization rate of PEGMA and found that the catalytic activity of iron complex could be markedly enhanced by the introduction of electron donating groups into the TPP ligand.

Yamamoto and coworkers found that the iron mediated ATRP with TPP as a ligand could be successfully applied to grafting methyl methacrylate to polyethylene (PE) [109]. Ying et al. conducted an iron mediated ATRP of AN with TPP as a ligand [110]. Noh and his colleagues performed iron mediated ATRP of styrene and MMA using DPPP and DPPMP as ligands and showed that FeBr_2_/DPPP had high catalytic activity in the ATRP of MMA but poor catalytical performance in the ATRP of styrene [106]. In order to promote the polymerization rate of iron mediated ATRP, Matyjaszewski et al. investigated the iron mediated ATRP of styrene using a series of phosphines such as TPP, TMPP, and TTMPP as ligands [86]. They revealed that Fe(III)X_3_/TTMPP was very active in the ATRP of styrene, and a high monomer conversion of 92% could be achieved in 21 h. Noh and coworkers found that the iron mediated ATRP of MMA using TPP, DPPP, or TEP as a ligand was well controlled, but the polymerization of styrene showed poor controllability [82]. They later also investigated iron mediated ATRP using 2-(diphenylphosphino)-*N,N*′- dimethyl-[1,1′-biphenyl]-2-amine (DPPDMA), DPPB, DPPBA, DPMPP, BDPPM, BDPPE, and 1,3-bis(diphenylphosphino)propane (BDPPP) as ligands [74,80,83].

#### 2.7.3. Organic Acid-Based Ligands

The nitrogen-based ligands and the phosphorous ligands have been widely used in iron mediated ATRP. However, many these ligands are practically toxic and harmful to human health. Therefore, finding ‘green’ ligands for iron mediated ATRP attracts considerable attentions of chemical researchers. Organic acids seem to be excellent ligands for iron mediated ATRP because of their low toxicity and inexpensive cost characteristics. These acids include pyromellitic acid [48], IDA [111], succinic acid (SA) [62], acetic acid [112], isophthalic acid (IA) [113], and ethylenediamine tetraacetic acid (EDTA) [114].

Yan and coworkers carried out an iron mediated ATRP of styrene using SA as ligand and obtained polystyrene with controlled molecular weights and low polydispersities (*M*_w_/*M*_n_ = 1.23–1.53) [115]. The iron mediated ATRP of MMA with IA as ligand and ethyl 2-bromopropionate (EBP) as initiator was found to be well controlled in polar solvents such as *N*,*N*-dimethylformamide [113]. However, the polymerization could not be successfully finished in bulk or in nonpolar solvents because the solubility of the catalyst and ligand in monomer or nonpolar solvents were very limited.

Ji and coworkers reported an iron mediated ATRP of acrylonitrile and prepared a poly(acrylonitrile)-*g*-poly(styrene) copolymer using IDA as ligand [116]. The copolymer could be further modified by NH_2_OH·HCl to produce amidoxime poly(acrylonitrile)-*g*-poly(styrene) beads, which had an excellent adsorption selectivity for Hg^2+^. Similar poly(acrylonitrile) had also been synthesized with IDA as ligand by Chen group in 2011 [117]. Hou et al. used iron mediated ATRP of acrylonitrile with IDA or SA as ligand to yield poly(acrylonitrile) (PAN) with well-controlled molecular weights [118,119]. Recently, an iron mediated ARGET ATRP of AN using IA as ligand and VC as reducing agent was reported by Chen group, and PAN with narrow molecular weight distributions (*M*_w_/*M*_n_ = 1.14–1.38) were obtained [120]. Iron mediated reverse ATRP of MMA with pyromellitic acid as ligand had been explored by Zhu and coworkers in 2003 [48]. A series of (di)picolinic acids including 2,6-pyridine dicarboxylic acid (PDA 1), 2,3-pyridine dicarboxylic acid (PDA 2), 3,5-pyridine dicarboxylic acid (PDA 3), 2,5-thiophene dicarboxylic acid (PCA), and their derivatives have been developed as ligands for iron mediated ATRP in their group [121]. They concluded that the polymerization rate and the polymer polydispersity were largely depended on the structure of ligands.

EDTA was also an excellent ligand for iron mediated ATRP. Malmström and coworkers reported an iron mediated ATRP of styrene using EDTA as ligand [122]. Similarly, Wu and coworkers reported a Fe(0) mediated single electron transfer-living radical polymerization (SET LRP) of MMA using EDTA as ligand and obtained PMMA with controlled molecular weights and low polydispersities (*M*_w_/*M*_n_ = 1.19–1.35) in the presence of a limited amount of air [123].

#### 2.7.4. Onium Salt-Based Ligands

Onium salt is also a type of widely used ligand in iron mediated ATRP. Matyjaszewski and coworkers explored a large variety of onium salts as iron ligands and found that FeBr_3_/onium salts catalyzed well-controlled reverse ATRP of MMA and MA, but the catalysts were not efficient for the polymerization of styrene [32]. Zhu et al. investigated the iron mediated ATRP of styrene using onium salt as ligand and discussed the effects of different onium salts such as tetrabutylammonium triflate (TBAOTf), triphenylamino phosphonium bromide (TPAPB), dimethyl diallylammonium chloride (DMDAAC), hexadecyl trimethyl ammonium chloride (HDTMAC), and hexadecyl trimethyl ammonium bromide (HDTMAB) on the polymerization rate and polydispersities of obtained polymers [124].

Matyjaszewski group investigated iron mediated ATRP of MMA in the presence of a series of salts including TBAOTf, tetrabutylammonium perchlorate (TBAClO_4_), tetrabutylammonium with BF_4_ anion (TBABF_4_), and tetrabutylammonium with PF_6_ anion (TBAPF_6_) [125]. They found that iron/TBAOTf could catalyze ATRP of MMA with excellent controllability not only in anisole but also in nonpolar solvents. The weakly coordinating triflate anions were beneficial to the dissolution of Fe^II^Br_2_. The iron mediated ATRP of MMA with phosphazenium salts (PZN-X; X = Cl, Br, I) as ligands had been studied by Inoue and coworkers in 2009 [126]. They found that the phosphazenium halide was an excellent cocatalyst, and the in situ formed iron halide/phosphazenium halide complex had an excellent catalytic performance in ATRP of alkyl and functionalized methacrylates.

Ionic liquids (ILs) have been used in iron mediated ATRP due to their low volatility and high stability. A series of ILs such as 1-butyl-3-methylimidazolium bromide (MIBR), 1-butyl-3-methylimidazolium chloride (MICH), 1-butyl-3-methylimidazolium dodecyl sulfate (MICDDS), and 1-butyl-3-methylimidazolium carbonate (MICar) have been investigated by Matyjaszewski et al. in iron mediated ATRP [127]. The polymerization produced PMMA with controlled molecular weights and narrow molecular weight distributions (*M*_w_/*M*_n_ = 1.24–1.55) in the presence of ILs without any other organic ligands. The iron mediated reverse ATRP of MAN in the presence of ionic liquids such as 1-methylimidazolium acetate ([mim][AT]), 1-methylimidazolium caproate ([mim][CT]), 1-methylimidazolium butyrate ([mim][BT]), and 1-methylimidazolium heptylate ([mim][HT]) had been inspected by Chen and coworkers [128]. A poly(methacrylonitrile)-*b*-poly(styrene) block copolymer had been synthesized in the presence of 1-methylimidazolium acetate using PMAN as a macroinitiator.

#### 2.7.5. Miscellaneous Ligands

Generally, iron ions could complex with any ligand that has a coordination site. Therefore, a number of polar solvents such as acetonitrile (MeCN) and *N*,*N*-dimethylformamide (DMF) may be potential ligands for iron mediated ATRP [129,130].

Xue and coworkers conducted iron mediated ATRP of MMA in polar solvents such as *N*-methylpyrrolidone, DMF, and MeCN using Sn(EH)_2_ as a reducing agent [129]. They studied the effects of solvents and different initiators on the polymerization of MMA and found that most of the polymerizations showed excellent controllability. Xue et al. later also succeeded in iron mediated ATRP of MMA in polar solvents using alcohols (e.g., methanol, ethanol, ethylene glycol, glycerol) as reducing agents [130].

As a summary, Table 1 lists representative experimental data of iron complex catalyzed ATRP. A large variety of iron compounds coordinated with nitrogen (Table 1, Entry 3, 11, 16, 24, 34, 46, 47, and 81), phosphorus (Table 1, Entry 4, 5, 25, 28, 36, 53–56, 76, and 78), organic acid (Table 1, Entry 21, 37, 38, and 40), onium salt ligands (Table 1, Entry 9, 10, 41, 48, and 63) have been employed as ATRP catalysts for controlled polymerizations of acrylate (Table 1, Entry 22–24, 79, 81, and 82), methacrylate (Table 1, Entry 3–5, 9, 10, 27–29, 42–46, 50–56, 63, 70–74, and 80), styrene (Table 1, Entry 11, 14, 16, 18, 47, 48, 64–66, and 75–78), and acrylonitrile (Table 1, Entry 36–38) due to their low toxicity, high abundance, and environmental friendliness. A large number of well-defined homo- (Table 1, Entry 3–5, 9–11, 22–25, 27–29, 36–43, 46–48, 50–56, 63–65, 70–72, 74–83), block- (Table 1, Entry 4, 9, 23, 25, 27–29, 36, 40–43, 46, 47, 50, 51, 63, 71, 82, 83), and graft copolymers (Table 1, Entry 26) have been successfully synthesized via iron mediated ATRP.

## 3. The Enzyme Mediated ATRP System

Though iron mediated ATRP has been successfully developed and applied to controlled polymerization of a large number of vinyl monomers in recent two decades, the residual iron catalyst in polymer products still presents a challenge to the industrialization of iron mediated ATRP and practically limits its application in biomaterials or microelectronics. Enzymes are usually non-toxic, highly selectable, biodegradable, and environmentally friendly biocatalysts and have been used to synthesize a large number of polymers under mild reaction conditions due to their biocompatible characteristic and high catalytic efficiency. Similarly, enzymes have been utilized as highly efficient catalysts in ATRP system.

Di Lena and coworkers reported that the laccase derived from fungus *Trametes versicolor* (LTV) could induce ATRP of methacrylic monomers in the presence of alkyl halide initiators and VC [131]. A couple of alkyl halides including EBiB, 2-bromopropionitrile (BPN), ethyl iodoacetate (EIAc), methyl 2-chloropropionate (MCP), and 2-cyano-2-propyl dithiobenzoate (CPDB) were explored in the polymerization of PEGMA. Among these initiators, BPN showed the highest initiation efficiency and produced corresponding polymers with relatively low polydispersity index (PDI = 1.94). The catalytical system was also applied to the polymerization of hydrophobic monomers such as MMA with EBiB as an initiator in the presence of LTV. Later, di Lena et al. performed the polymerization of poly(ethylene glycol) methyl ether acrylate (PEGA) using catalase derived from bovine liver (CBL) as catalyst [132]. The molecular weights of obtained poly(PEGA) increased linearly with the increase of monomer conversions when using BPN as an initiator, and the molecular weight distribution of poly(PEGA) was relatively narrow (PDI = 1.60). Moreover, the polymerization of PEGA in the presence of LTV or horseradish peroxidases (HRP) was also conducted, and the molecular weight of poly(PEGA) was found to increase with the increase of monomer conversion.

Bruns et al. conducted the ATRP of *N*-isopropylacrylamide (NIPAAm) using HRP as a catalyst and 2-hydroxyethyl-2-bromoisobutyrate (HEBiB) as an initiator and prepared poly(NIPAAm) (PNIPAAm) with a high molecular weight (*M*_n_ = 9.99 × 10^4^ Da) and low polydispersity index (PDI = 1.44) [133]. The polymerization kinetics and the effect of different pH (5.2 to 10.5) on the polymerization were investigated. It was found that the highest monomer conversion (78%) was obtained at pH = 7.0. Bruns and coworkers also reported other enzymes (e.g., hemoglobin and red blood cells) mediated ATRP of NIPAAm, PEGMA, and PEGA using HEBiB as an initiator and VC as a reducing agent [134]. All the polymerizations showed a first order kinetic characteristic but a relatively poor controllability. Only the polymerization of PEGA using BPN as an initiator produced poly(PEGA) with a low polydispersity index (PDI < 1.11). The polymerization of PEGA in a polymersome, poly(dimethylsiloxane)-*block*-poly(2-methyl-2-oxazoline), using HRP as a catalyst and HEBiB as an initiator had also been reported by their group [135]. Kadokawa and coworkers carried out ATRP of NIPAAm using an enzyme mimetic (hematin) as catalyst and found that the number average molecular weight (*M*_n_) of the synthesized poly(NIPAAm) increased linearly with the increase of monomer conversion, but the molecular weight distribution of poly(NIPAAm) was relatively broad (PDI = 1.8–2.1) [136].

Matyjaszewski and coworkers reported polymerization of oligo(ethylene oxide) methyl ether methacrylate (OEOMA) using hemin or its modified products [hemin-(PEG_1000_)_2_ and mesohemin-(MPEG_550_)_2_, MPEG = methoxy PEG] as catalyst [137]. The hemin mediated ATRP showed poor controllability due to its low halidophilicity. The controllability could be improved by using hemin-(PEG_1000_)_2_ as catalyst. Correspondingly, the obtained poly(OEOMA) had a low polydispersity index (PDI = 1.32) and its molecular weight increased linearly with the increase of monomer conversion. The polymerization could produce poly(OEOMA) with more narrow molecular weight distribution (PDI = 1.19) when mesohemin-(MPEG_550_)_2_ was used as a catalyst.

Deuterohemin-β-Ala-His-Thr-Val-Glu-Lys (DhHP-6) is a synthesized heme-containing peroxidase mimic showing high catalytic performance. Tang and coworkers carried out the DhHP-6 mediated ARGET ATRP of PEGMA and GMA [138]. They found that the molecular weights of corresponding polymers increased linearly with the increase of monomer conversions. Well-defined poly(PEGMA) (*M*_n_ = 6.02 × 10^3^ Da, PDI = 1.08) and poly(GMA) (*M*_n_ = 8.43 × 10^3^ Da, PDI = 1.38) have been successfully synthesized. Poly(ε-caprolactone) (PCL) had been synthesized via enzymatic ring-opening polymerization (eROP) using novozyme 435 as a catalyst and HEBiB as an initiator [138]. The synthesized PCL-Br was further used as a macroinitiator to prepare amphiphilic copolymers such as PCL-PHEMA and PCL-PMAA (PMAA = poly(methacrylic acid)) using DhHP-6 as a catalyst. The integration of eROP and enzyme mediated ATRP was a promising environmentally benign process for the preparation of biomaterials. Tang et al. later reported the polymerization PEGMA using DhHP-6@ZIF-8 (DhHP-6@ZIF-8 = DhHP-6 embedded in zeolite imidazolate framework-8) as a catalyst and BPN as an initiator [139]. This catalyst could be applied to the polymerization of PEGA and NIPAAm.

As oxygen is an undesirable radical inhibitor, it is important to develop a polymerization system that can be conducted in oxygen-rich environments. Matyjaszewski and coworkers reported a well-controlled aqueous ATRP conducted in the open air [140]. This ATRP was realized by continuous conversion of oxygen (O_2_) to carbon dioxide (CO_2_) using glucose oxidase (GOx) as catalyst in the presence of sodium pyruvate. In the first step of the polymerization, the glucose and oxygen were converted into d-glucono-1,5-lactone and hydrogen peroxide (H_2_O_2_). Therefore, the inhibition of O_2_ to the polymerization was eliminated. Then, the toxic H_2_O_2_ produced in the first step was consumed via the reaction between H_2_O_2_ and sodium pyruvate to yield CO_2_, water and acetate. In this case, the ATRP of OEOMA could be successfully achieved, producing poly(OEOMA) with low dispersity (1.09 ≤ PDI ≤ 1.29). In addition, block copolymer poly(OEOMA)-*b*-poly(OEOMA) (*M*_n_ = 7.94 × 10^4^ Da, PDI = 1.28) had also been prepared by using poly(OEOMA) (*M*_n_ = 4.23 × 10^4^ Da, PDI = 1.23) as a macroinitiator. This GOx mediated approach was also used to polymerize BA and BMA using ethyl α-bromophenylacetate (EBPA) as an initiator, and well-defined poly(BA) (*M*_n_ = 2.53 × 10^4^ Da, PDI = 1.24) and poly(BMA) (*M*_n_ = 3.24 × 10^4^ Da, PDI = 1.16) have been successfully prepared [141].

Matyjaszewski et al. also reported HRP mediated ATRP of OEOMA using α-bromophenylacetic acid (BPAA) as an initiator [142]. The effect of HRP concentration on the polymerization was investigated. It was found that the polymerization rate increased with the increase of HRP concentration. The monomer conversion reached 94% in 30 min at a HRP concentration of 1130 nM, producing poly(OEOMA) with a low polydispersity index (PDI = 1.17). The chain extension was realized by using poly(OEOMA_500_)-Br (*M*_n_ = 3.82 × 10^4^ Da, PDI = 1.13) as a macroinitiator to copolymerize with OEOMA_300_. Moreover, this system was also applied to the copolymerization of OEOMA with bovine serum albumin (BSA) or human serum albumin (HSA). Well-defined BSA-*b*-poly(OEOMA) (*M*_n_ = 6.31 × 10^4^ Da, PDI = 1.38) and HSA-*b*-poly(OEOMA) (*M*_n_ = 4.01 × 10^4^ Da, PDI = 1.25) bioconjugates have been synthesized.

Polymer brush can be used as surface coating due to its ability to endow an interface with a large number of useful properties such as biocompatibility [143], lubrication and protein-resistance [144,145]. The surface-initiated atom transfer radical polymerization (SI-ATRP) is a commonly used approach for the preparation of polymer brush. Zauscher and coworkers reported the synthesis of biomedically relevant polymer brushes such as poly(oligo(ethylene glycol) methacrylate) (POEGMA), poly(sulfobetaine methacrylate) (PSBMA), poly(2-dimethylaminoethyl methacrylate) (PDMAEMA), and poly(2-(methylsulfinyl)ethyl acrylate) (PMSEA) via enzyme (GOx) mediated SI-ATRP in an open air environment [146]. The presence of GOx improved the fouling resistance of the polymer materials. Bruns et al. found that the enzyme mediated surface-initiated biocatalytic atom transfer radical polymerization (SI-bioATRP) could be used to prepare PNIPAAm brushes [147]. This method provided a new way for the translation of bioadhesion into a controlled functionalization of materials.

Enzyme catalyzed ATRP has attracted considerable attentions due to the high efficiency, excellent selectivity, mild reaction conditions, and good biocompatibility of enzymes. Representative experimental data of enzyme catalyzed ATRP are summarized in Table 2. The polymerizations show typical ‘living’/controlled characteristics and produces a lot of well-defined homo- (Table 2, Entry 4, 6, 11, 12, and 15–18), block- (Table 2, Entry 1, 5, and 17) and brush polymers (Table 2, Entry 19) with good water-solubility and biocompatibility.

## 4. The Metal-Free Catalyst Mediated ATRP System

Enzyme mediated ATRP has the advantages of high efficiency, mild reaction conditions, and synthesizing biocompatible polymers, which have potential valuable applications in materials science and biomedical engineering areas. However, it suffers from the problems of narrow range of polymerizable monomers and less applicable enzymes. Chemists always envisage developing metal-free catalyst and constructing new environmentally friendly technology for ATRP. The organic photoredox catalyst has been proved to be an excellent candidate [148,149].

### 4.1. Organic Photocatalyst Mediated Metal-Free ATRP

#### 4.1.1. Phenothiazines Mediated ATRP

Hawker and coworkers firstly reported in 2014 a photoinduced metal-free ATRP of methacrylates using 10-phenylphenothiazine (Ph-PTZ) as an organic photocatalyst under UV light irradiation (380 nm) [149]. The polymerization showed good controllabilities for MMA, benzyl methacrylate (BnMA), and DMAEMA. The produced PMMA, poly(benzyl methacrylate) (PBnMA) and PDMAEMA had narrow molecular weight distribution of 1.18, 1.25, and 1.11 respectively. Well-defined block copolymer PMMA-*b*-PBnMA (*M*_n_ = 2.59 × 10^4^ Da, PDI = 1.31) was also prepared via metal-free ATRP by using PMMA as a macroinitiator and Ph-PTZ as a photocatalyst.

Matyjaszewski group performed a photoinduced metal-free ATRP of AN using Ph-PTZ as an organic photocatalyst and EBPA as an initiator, and obtained PAN with *M*_n_ = 6.20 × 10^3^ Da and PDI = 1.60 [150]. They also tried the polymerization of AN using 10-(4-methoxyphenyl)-phenothiazine (4-MeOPh-PTZ) or 10-(1-naphthalenyl)-phenothiazine (Nap-PTZ) as a photocatalyst and BPN as an initiator. The molecular weights of produced PAN increased with the increase of monomer conversion at the early stage of the polymerization, but were much higher than theoretical values, indicating that the initiation efficiency of BPN was lower than that of EBPA.

Matyjaszewski et al. also conducted a metal-free ATRP of MMA using Ph-PTZ as a photocatalyst and EBPA as an initiator [151]. The produced PMMA had a molecular weight (*M*_n,GPC_ = 2.07 × 10^3^ Da) close to its theoretical value (*M*_n,th_ = 1.80 × 10^3^ Da) and the molecular weight distribution was relatively narrow (PDI = 1.50). A number of photocatalysts including 10-methylphenothiazine (Me-PTZ), benzo[b]phenothiazine (Ph-benzoPTZ), 9-phenylcarbazole (Ph-CBZ), thianthrene (TH), and *N*-aryl phenothiazine derivatives (Nap-PTZ) have been investigated in the polymerization of MMA. The results indicated that the Ph-benzoPTZ and Nap-PTZ mediated polymerizations showed better controllability than Ph-PTZ. Moreover, a mechanism investigation of the polymerization catalyzed by phenothiazine derivatives revealed that all the selected catalysts were involved in the activation step, but only part of them were efficiently participated in the deactivation step, leading to different controllability of the polymerization.

Tran et al. reported metal-free ATRP of methacrylate monomers such as MMA, HEMA, and DMAEMA using 4-(10H-phenothiazin-10-yl)-*N*,*N*-diphenylaniline (PDPA) as a photocatalyst and phenyl 2-bromo-2-methylpropionate (PhBMP) as an initiator under UV irradiation [152]. The polymerization reached a high monomer conversion of 94.6% and produced PMMA with molecular weight close to theoretical value, indicating that the polymerization was controlled. They also investigated the influence of solvents on the polymerization of MMA and found that the polymerization performed in THF gave higher yield (94.6%) than in DMF (85%) and toluene (43%).

#### 4.1.2. Aromatic Hydrocarbons Mediated ATRP

Miyake and coworkers developed a metal-free ATRP of methacrylates using perylene as an organic photocatalyst and EBPA as an initiator and prepared PMMA and PBA with narrow molecular weight distributions (PDI < 1.30) [153]. The catalyst system had also been successfully applied to the preparation of PMMA-*b*-PMMA (*M*_w_ = 3.43 × 10^5^ Da, PDI = 1.45), PMMA-*b*-PS (1.65 × 10^5^ Da, PDI = 1.39), and PMMA-*b*-PBA (*M*_w_ = 5.23 × 10^5^ Da, PDI = 2.55) block polymers.

Yilmaz group later reported metal-free ATRP of methacrylates and other vinyl monomers using pyrene or anthracene as catalyst and alkyl halides such as EBiB, 1-bromoethyl benzene (BEB), EBP as initiators [154]. They found that EBiB and BEB showed higher initiation efficiency than EBP in the polymerization of MMA and produced PMMA with lower polydispersity index (PDI = 1.38, 1.37 respectively). Other vinyl homopolymers such as poly(tert-butyl acrylate) (*M*_n_ = 1.07 × 10^5^ Da, PDI = 1.32) and PS (*M*_n_ = 2.00 × 10^3^ Da, PDI = 1.32) and PMMA-*b*-PMMA (*M*_n_ = 1.92 × 10^5^ Da, PDI = 1.40) as well as PMMA-*b*-PS (*M*_n_ = 1.85 × 10^4^ Da, PDI = 1.50) block copolymers had also been prepared using the same catalyst system.

#### 4.1.3. Fluorescein Mediated ATRP

Fluorescein has good chemical stability, visible region absorbance, and favorable redox potential, and can activate alkyl bromide and induce metal-free ATRP by a reductive quenching pathway in the presence of electron donors. Zhang et al. reported a metal-free ATRP of MMA using fluorescein (FL) as an organic photocatalyst in the presence of triethylamine (TEA) [155]. The polymerization was controlled and produced PMMA with relatively narrow molecular weight distribution (PDI = 1.46). In order to expand the scope of monomer, styrene, GMA, PEGMA, BnMA, and AN have been polymerized using fluorescein (FL) as a photocatalyst. These polymerizations presented lower controllability than MMA.

Yagci and coworkers conducted metal-free ATRP of MMA using eosin Y or erythrosin B as a photocatalyst in the presence of electron donor amines [156]. The polymerization was completed using EBP as initiator and PMDETA as electron donor under visible light irradiation. The results demonstrated that eosin Y and erythrosin B had higher catalytical activity and controllability than fluorescein and could produce PMMA with narrow molecular weight distributions (PDI = 1.33, 1.20 respectively). The system was also applied to the homopolymerization of other vinyl monomers including styrene, tert-butyl acrylate (t-BA), and HEMA and block copolymerization to prepare PMMA-*b*-PMMA (*M*_n_ = 2.27 × 10^4^ Da, PDI = 1.41) and PMMA-*b*-PS (*M*_n_ = 2.79 × 10^4^ Da, PDI = 1.60) block copolymers.

#### 4.1.4. Phenazines and *N*-aryl Phenoxazines Mediated ATRP

Miyake et al. reported metal-free ATRP of MMA using dihydrophenazine derivative as a photocatalyst and EBPA as an initiator under the irradiation of white LEDs [157]. The dihydrophenazine derivatives are a kind of new efficient visible light stimulating photocatalyst possessing high excited state reduction potential (*E*^0^* = −2.36 to −2.06 V). The effect of different dihydrophenazine derivatives on the polymerization has been investigated, showing that 5,10-di(4-trifluoromethylphenyl)-5,10-dihydrophenazine (PhenN-CF_3_) had more advantages in producing PMMA with a combination of the highest initiation efficiency (65.9%) and the lowest polydispersity index (PDI = 1.17). In addition, PhenN-CF_3_ mediated metal-free ATRP of MMA was also achieved under sunlight. The *M*_n_ of produced PMMA increased linearly with the increase of monomer conversion and the molecular weight distribution was very narrow (PDI = 1.10), indicating that the polymerization was well-controlled. Miyake and coworkers also synthesized a series of *N*-aryl phenoxazines and successfully used them as catalysts to mediate ATRP of MMA, isobutyl methacrylate (IBMA), BnMA, and isododecyl methacrylate (IDMA) [158].

#### 4.1.5. Carbazoles Mediated ATRP

Zhang and coworkers performed a metal-free ATRP of MMA using 1,2,3,5- tetrakis(carbazol-9-yl)-4,6-dicyanobenzene (4CzIPN) as a photocatalyst and EBPA as an initiator under the irradiation of blue light emitting diode [159]. The effect of photocatalyst concentration (5 to 1500 ppm) on the polymerization has been investigated. It was found that the molecular weight distribution of PMMA became broader with the increase of photocatalyst concentration and the polymerization could be achieved without the initiator. The polymerization reached a high monomer conversion (90%) and was well-controlled even at a low concentration of photocatalyst (15 ppm), producing PMMA with a relatively narrow molecular weight distribution (PDI = 1.50). The initiation efficiency of the polymerization was as high as 95.2%.

#### 4.1.6. Benzaldehyde Derivative Mediated ATRP

Yang et al. reported that the metal-free ATRP of methacrylates could be mediated by benzaldehyde derivative photocatalyst [160]. Three benzaldehyde derivatives including p-anisaldehyde (*E*^0^* = −2.42 V), p-cyanobenzaldehyde (*E*^0^* = −2.19), and 2,4-dimethoxy benzaldehyde (*E*^0^* = −2.60) were investigated in the polymerization. The effects of different initiator including EBiB, EBPA, and perfluoro-1-iodohexane (CF_3_(CF_2_)_5_-I) on the polymerization have been studied. The results showed that the polymerization of PEGMA could be controlled by using p-anisaldehyde as a catalyst while the polymerization of MMA could be controlled by using p-cyanobenzaldehyde as a catalyst. The polymerization of MMA showed a longer induction period due to the relatively higher oxidation reduction potential of p-cyanobenzaldehyde. The effect of initiator concentration on the polymerization of PEGMA was investigated using 2,4-dimethoxy benzaldehyde as a catalyst and (CF_3_(CF_2_)_5_-I) as an initiator. Well-defined homopolymer poly(PEGMA) (PPEGMA) (*M*_n_ = 1.33 × 10^4^ Da, PDI = 1.21) and block copolymers including PBnMA-*b*-PMMA (*M*_n_ = 2.23 × 10^4^ Da, PDI = 1.92), PPEGMA-*b*-PMMA (*M*_n_ = 6.45 × 10^4^ Da, PDI = 1.87), and PPEGMA-*b*-PBnMA (*M*_n_ = 5.48 × 10^4^ Da, PDI = 2.29) have been synthesized.

#### 4.1.7. Other Photocatalyst Mediated ATRP

Wang et al. conducted metal-free ATRP of methacrylates under visible LED light irradiation [161]. *N*,*N*-bis(tert-butyloxycarbonyl)-quinacridone (TBOC-QA), *N*,*N*-bis(tert-butyloxycarbonyl)- thiophenediketopyrrolopyrrole (TBOC-DPP), and *N*,*N*-bis(tert-butyloxycarbonyl)-indigo (TBOC-Indigo) were developed as organic photocatalysts. The effects of fluorescence quantum yield, photostability, and reduction potential of these photocatalysts on the polymerization were investigated by using MMA as a monomer and alkyl bromide as an initiator. They found that the polymerization with TBOC-QA as photocatalyst showed excellent controllability, but the polymerization with TBOC-DPP as photocatalyst had a low initiation efficiency of 6.5% due to the poor photostability and electrochemical stability of TBOC-DPP. TBOC-indigo had the lowest fluorescence quantum yield and did not initiate any polymerization. These results indicated that TBOC-QA was a promising photocatalyst for light-controllable ATRP. To investigate the scope of polymerizable monomers in TBOC-QA mediated ATRP, other vinyl monomers including BMA, 2-(diisopropylamino) ethyl methacrylate (DPA), styrene, or OEGMA were also tested. All the monomers except styrene could be well polymerized with TBOC-QA as a photocatalyst.

### 4.2. Applications of Metal-Free ATRP in The Preparation of Composite Materials

Amphiphilic block copolymer attracts considerable attention due to its self-assemble ability, and has been widely used in drug and gene delivery areas [162,163,164,165]. Son et al. reported that an amphiphilic diblock copolymer could be prepared through metal-free ATRP by using Ph-PTZ as organic photocatalyst under the irradiation of LED light (380 nm) [166]. This method was also successfully applied to the polymerization of MMA, GMA, BA, 2-diethylaminoethyl methacrylate (DEAEMA), and allyl methacrylate (AMA). The PMMA-*b*-PBA (*M*_n_ = 7.5 × 10^3^ Da, PDI = 1.50) block copolymer and poly(ethylene glycol)-*b*-poly(glycidyl methacrylate) (PEG-*b*-PGMA) amphiphilic block copolymer were prepared by using PMMA-Br and PEG-Br respectively as a macroinitiator and Ph-PTZ as a photocatalyst. The epoxide groups of PEG-*b*-PGMA could react with polyethylenimine to produce a cationic polymer bearing oligoamine side chains, which could be applied to gene delivery [167]. They later investigated visible light-mediated metal-free ATRP of MMA using *N*-trifluoromethylphenyl phenoxazine derivatives as organic photocatalysts [168]. They found that the polymerization was significantly affected by the visible light absorption efficiency, excited state reduction potential, and spatially separated singly occupied molecular orbitals (SOMOs) of the catalyst. The visible light absorption could be enhanced by introducing a biphenyl ring or phenyl with electron-withdrawing groups into the phenoxazine core. On the other hand, a strong excited state reduction potential and spatially separated SOMOs were beneficial for preparing well-defined polymers.

SiO_2_ hollow spheres (HS) have attracted much attention due to their wide potential applications in electrical materials and catalysis [169], biomacromolecule delivery [170], controlled drug-release carriers [171], and optical devices [172]. However, the application of SiO_2_ HS is largely limited because of its low physical loading ability and poor hydrophobicity. Therefore, surface modification of SiO_2_ HS to improve its solubility and physicochemical properties is essential to expand its application scope. Wang and coworkers prepared an amphiphilic diblock copolymer poly(methyl methacrylate)-*b*-poly(*N*-isopropylacrylamide) grafted HS (HS-*g*-PMMA-*b*-PNIPAAm) hybrid material via metal-free surface-initiated ATRP using Ph-PTZ as a photocatalyst and α-bromoisobutyryl bromide (BIBB) as an initiator [173]. They investigated the dispersions of HS, HS-*g*-PMMA, and HS-*g*-PMMA-*b*-PNIPAAm in inorganic (H_2_O) and organic solvent (THF) and found that the HS was dispersed in water but aggregated in THF while the HS-*g*-PMMA showed a complete opposite dispersibility to HS, indicating that the grafted PMMA improved the surface hydrophobicity of HS. The HS-*g*-PMMA-*b*-PNIPAAm could be dispersed in both THF and H_2_O, implying that the PNIPAAm chains were beneficial for the increase of surface amphiphilicity.

Wang et al. later prepared poly(DEAEMA) (PDEAEMA) grafted silica nanoparticles (SNPs) (SNPs-*g*-PDEAEMA) and used them for quercetin (Qu) controlled-release [174]. The SNPs-*g*-PDEAEMA was a kind of pH-sensitive material which was dispersed in acid but aggregated in neuter and alkaline solutions. The self-assembled Qu-loaded microcapsules formed a tight structure under normal physiological conditions (pH = 7.4) with drug entrapped in the core, but the microcapsules became swollen under a weak acid owing to the protonation of the amine groups of PDEAEMA, resulting in drug release from the inner cores. They also evaluated the in vitro cytotoxicity of SNPs-*g*-PDEAEMA to L929 cells and found that the cell viabilities kept 90.18% and 92.43% on the first and second day respectively, and the cytotoxicity was completely disappeared on the third day. These results showed that the SNPs-*g*-PDEAEMA had excellent biocompatibility and could be served as drug carriers.

Cellulose is a kind of abundant, inexpensive and renewable biopolymeric materials, and is largely used in daily life. Compared to synthetic polymers derived from petroleum resources, cellulose shows poor solubility in organic solvents, low dimensional stability, and insufficient crease resistance. Wang and coworkers reported a method to prepare cellulose-grafted copolymers using biomass-based monomers such as lauryl methacrylate (LMA), furfuryl methacrylate (FMA), and rosin monomer (DAGMA) via photoinduced metal-free ATRP with Ph-PTZ as a photocatalyst and bromated ethyl cellulose (EC-Br) as an initiator [175]. A series of EC grafted copolymers including EC-*g*-PLMA (*M*_n_ = 1.67 × 10^4^ Da, PDI = 1.66), EC-g-PFMA (*M*_n_ = 1.20 × 10^4^ Da, PDI = 1.74), and EC-*g*-PDAGMA (*M*_n_ = 1.84 × 10^4^ Da, PDI = 1.79) have been synthesized.

Wang et al. reported a recyclable and sustainable flexible thermoset elastomer derived from fatty acid, furfural, and cellulose via the combination of metal-free ATRP with Diels–Alder reaction [176]. Firstly, the thermoplastic ethyl cellulose (EC) grafted copolymer, EC-*g*-poly(lauryl methacrylate-*co*-furfuryl methacrylate) (EC-*g*-P(LMA-*co*-FMA)), was synthesized via metal-free ATRP using Ph-PTZ as catalyst. Then, a modified epoxidized soybean oil containing 6-maleimidohexanoic group (ESOM) was employed to conduct Diels–Alder reaction with furfural groups in the chain of EC-*g*-P(LMA-*co*-FMA) to prepare the recyclable thermoset elastomers. The formation of dynamic crosslinking structure by Diels–Alder reaction provided an excellent self-healing and recyclability for the thermoset elastomers.

Hydrogels have been widely applied in the fields of drug-controlled release [177], biosensors [178], tissue engineering and adsorbents [179,180]. However, the service life of conventional hydrogel is short due to its poor mechanical strength. The mechanical strength and self-healing properties of hydrogel can be enhanced by cellulose nanocrystals (CNCs) due to their highly crystalline and nontoxic nanorods characteristics [181]. Bai et al. reported the preparation of self-healing nanocomposite hydrogels based on modified CNCs [182]. 4-vinylpyridine (4VP) was surface-initiated onto the surface of CNCs via metal-free ATRP using Ph-PTZ as a photocatalyst to form poly(4-vinylpyridine) CNCs hybrid material (CNCs@P4VP). The CNCs@P4VP was an excellent reinforcement for self-heal poly(acrylic acid) (PAA) hydrogels. The reversible electrostatic interaction between the carboxyl group of PAA and the pyridyl group of CNCs@P4VP acted as a dynamic reversible supramolecular interaction to heal and crosslink the PAA hydrogels. The prepared nanocomposite hydrogels showed an excellent self-healing (85.9% after repairing 6 h) and mechanical properties (6.6 MPa at a strain of 921.6%).

Table 3 summarizes typical experimental data of metal-free catalyst mediated ATRP. A large number of organic compounds including phenothiazines (Table 3, Entry 1, 3, 4, and 8), polynuclear aromatic hydrocarbons (Table 3, Entry 9 and 10), fluorescein (Table 3, Entry 11), *N*-aryl phenoxazines (Table 3, Entry 12), carbazoles (Table 3, Entry 13), and benzaldehyde derivative (Table 3, Entry 14), have been developed as photocatalyst and a lot of homopolymers [149], block polymers [183], star polymers [184,185], hyperbranched polymers [186], and composite materials [187,188,189] have been prepared via metal-free ATRP.

## 5. Summary and Future Perspectives

Though copper complex is the most commonly used ATRP catalyst, the application of copper catalyst at industrial scale is restricted due to its biological toxicity and environmentally unsafety [7,78]. Using iron complex as ATRP catalyst has attracted considerable interest in recent decades due to the negligible toxicity, low cost, and environmental friendliness of the iron catalyst. This review summarized the applications of iron catalyst in normal ATRP, reverse ATRP, ICAR ATRP, AGET ATRP, GAMA ATRP, and SARA ATRP in view of the catalytic activity, initiation efficiency, and polymerization controllability. A large variety of homo-, block-, graft-, brush-, star-, and hyperbranched polymers have been prepared via iron complex mediated ATRP and summarized in the review. As the catalytic activity of iron catalyst is largely depended on the ligand of the complex, the development of the catalyst ligand has also been discussed in this review.

Despite significant success and progress in iron complex-catalyzed ATRP, there are still challenges for iron catalysis. For instance, it is necessary to establish a structure–reactivity relationship for iron catalyst, especially the dependence of the activation and deactivation rate constant on the redox potential of iron complex. On the other hand, the polymerization of monomers containing strong polar functional groups is still troublesome due to the interaction between the polar groups and the iron catalyst. Therefore, developing more stable iron catalyst would be benefit to the development of iron-catalyzed ATRP. Moreover, developing new iron catalyst derived from biological resources for the preparation of biocompatible polymers would also be conducive to the iron complex catalyzed ATRP.

Though enzyme mediated ATRP has the advantage of synthesizing biocompatible polymers and bioconjugates, it suffers from limited polymerizable monomers and less applicable enzymes. It would be very advantageous to develop other enzymes such as hydrogenases and chlorophyl for ATRP. In addition, incorporating this method in more important reaction systems and fabricating multifunctional enzyme-containing materials with outstanding performances are also expected to be a direction of the enzyme mediated ATRP.

As metal-free ATRP is regarded as a green and sustainable process for precise polymer synthesis, a lot of desirable homo-, block-, star-, and hyperbranched polymers have been prepared via metal-free ATRP. However, a relatively large amount of photocatalyst is generally required in the polymerization. In addition, the use of photocatalyst often causes a problem of discoloration of the polymer products due to the highly colored performance of some photocatalysts. Further research is recommended to improve the catalyst activity, develop new photocatalysis strategy for cost-effective production of various polymeric materials and solve the problem of the discoloration. As many biocompatible materials such as HS-*g*-PMMA-*b*-PNIPAAm, SNPs-*g*-PDEAEMA, EC-*g*-PLMA, and CNCs@P4VP have been successfully prepared via metal-free ATRP, the application of metal-free ATRP in medicine, electricity, and other interdisciplinary areas would have a more promising perspective.

## Figures and Tables

**Table 1:** Iron complex catalyzed ATRP.

Entry	ATRP	Monomer	Iron	Ligand/Additive	Initiator	Catalyst Concentration(mmol/L)	Temp. (°C)	Time (h)	Conv. (%)	*M*_n_ (g/mol)	*M*_w_/*M*_n_	Ref.
1	Normal ATRP	MMA	FeCl_2_	TPP	CCl_4_^a^	10.0	80	30.0	90.0	5.31 × 10^3^	1.41	[26]
2		MMA	FeBr_2_	TnBP	(MMA)_2_Br ^b^	10.0	80	5.0	90.0	1.54 × 10^4^	1.42	[34]
3		MMA	FeBr_2_	NHPMI	EBiB	62.8	90	2.1	47.0	1.33 × 10^4^	1.21	[38]
4		MMA	FeBr_2_	DPPMP	BPN	23.4	90	5.0	83.0	1.70 × 10^4^	1.17	[40]
5		MMA	FeBr_2_	DMDPE	H-(MMA)_2_-Br ^c^	10.0	80	23.0	92.0	1.16 × 10^4^	1.25	[99]
6		MMA	FeCl_2_	MA_5_-DETA	EBiB	47.1	90	20.0	61.2	8.77 × 10^3^	1.29	[101]
7		MMA	FeCl_2_	DPDQMEDA	EBiB	31.6	90	1.5	87.5	2.60 × 10^4^	1.35	[104]
8		MMA	FeCl_2_	IA	EBP	18.8	90	10.0	89.0	5.41 × 10^4^	1.39	[113]
9		MMA	FeBr_2_	TBAOTf	EBPA	31.4	60	16.0	98.0	1.79 × 10^4^	1.20	[125]
10		MMA	FeBr_2_	MIBR	EBiB	125.4	60	7.3	65.3	8.60 × 10^3^	1.16	[127]
11		styrene	FeBr_2_	TnBA	PEBr	87.3	110	5.0	82.0	9.60 × 10^3^	1.13	[25]
12		styrene	FeCl_2_	acetic acid	CCl_4_^a^	43.5	120	23.0	75.0	1.47 × 10^4^	1.46	[35]
13		styrene	FeCl_2_	PMDETA	PECl	43.5	120	24.0	65.0	1.51 × 10^4^	1.62	[37]
14		styrene	FeBr_2_	TDA	PEBr	43.5	110	18.0	80.0	1.74 × 10^4^	1.20	[39]
15		styrene	FeBr_2_	DPPMP	PEBr	87.0	80	24.0	67.0	6.95 × 10^3^	1.41	[40]
16		styrene	FeCl_2_	Me_3_TACN	PECl	5.0	120	20.0	76.0	2.80 × 10^4^	1.20	[42]
17		styrene	FeBr_2_	(i-Pr)_3_TACN	PECl	34.9	120	4.0	95.0	2.60 × 10^4^	1.31	[43]
18		styrene	FeBr_2_	dNbpy	PEBr	34.9	110	21.0	64.0	6.47 × 10^3^	1.27	[103]
19		styrene	FeBr_2_	DPPP	PEBr	87.3	110	8.0	37.0	5.20 × 10^3^	1.42	[106]
20		styrene	FeCl_2_	SA	BEB ^d^	72.5	70	3.0	80.0	6.00 × 10^3^	1.30	[115]
21	Normal ATRP	styrene	FeCl_2_	EDTA	PEBr	5.6	50	0.5	32.0	3.10 × 10^3^	1.20	[122]
22		MA	FeBr_2_	TBPBr	EBP	47.7	90	23.2	32.0	6.40 × 10^3^	1.23	[32]
23		MA	Fe(Cp)I(CO)_2_	Al(Oi-Pr)_3_	(CH_3_)_2_C(CO_2_Et)I	40.0	60	80.0	93.0	1.21 × 10^4^	1.19	[33]
24		BA	FeBr_2_	(cyclopentyl)_3_TACN	EBiB	27.8	100	20.0	92.0	2.40 × 10^4^	1.24	[44]
25		PEGMA	FeBr_2_	TMPP	H-(MMA)_2_-Br ^c^	5.0	60	3.0	46.0	2.03 × 10^4^	1.14	[107]
26	Reverse ATRP	MMA	FeCl_3_	TPP	AIBN	55.4	90	8.0	76.1	1.56 × 10^5^	1.34	[109]
27		MMA	FeCl_3_	TPP	AIBN	29.0	85	2.0	85.0	7.50 × 10^4^	1.16	[45]
28		MMA	FeCl_3_	TPP	TPED	30.4	95	12.0	99.1	1.72 × 10^5^	1.13	[46]
29		MMA	FeCl_3_	pyromellitic acid	AIBN	25.1	100	6.0	88.6	2.78 × 10^4^	1.28	[48]
30		MMA	FeCl_3_	PDA 3	AIBN	31.4	100	18.0	93.0	2.16 × 10^4^	1.32	[121]
31		MMA	Fe(S_2_CN(C_4_H_9_)_2_)_3_	/	V-50	4.7	90	72.0	61.8	3.32 × 10^4^	1.34	[49]
32		styrene	FeCl_3_	BOX	TPED	23.3	120	20.0	N/A	2.10 × 10^4^	1.15	[50]
33		SMA	[Fe(DMF)_6_](ClO_4_)_3_	bpy	AIBN	1.0	80	N/A	N/A	1.48 × 10^5^	1.36	[53]
34		HMA	[Fe(DMF)_6_](ClO_4_)_3_	bpy	AIBN	20.0	80	N/A	N/A	7.75 × 10^4^	1.24	[54]
35		HEMA	FeCl_3_	TPP	BPO	N/A	80	24.0	90.0	2.37 × 10^4^	N/A	[55]
36		AN	FeCl_3_	TPP	TPED	15.0	70	6.0	48.3	5.32 × 10^3^	1.16	[110]
37		AN	FeCl_3_	IDA	AIBN	12.0	60	4.0	38.0	5.20 × 10^3^	1.16	[118]
38		AN	FeCl_3_	SA	AIBN	6.3	60	4.0	45.0	1.80 × 10^4^	1.17	[119]
39		DA	FeCl_3_	bpy	AIBN	1.0	80	N/A	50.0	1.20 × 10^4^	1.46	[57]
40		MAN	FeCl_3_	IA	AIBN	58.1	75	3.0	52.0	5.59 × 10^3^	1.13	[58]
41		MAN	FeCl_3_	[mim][AT]	AIBN	39.7	70	2.0	67.9	7.56 × 10^3^	1.23	[128]
42	ICAR ATRP	MMA	FeCl_3_	TPP	BMPB_2_	7.5	60	5.5	40.8	2.44 × 10^4^	1.24	[61]
43		MMA	FeCl_3_	SA	EBiB	0.6	90	36.0	36.4	2.12 × 10^4^	1.22	[62]
44		MMA	FeBr_3_	TBABr	EBPA	0.6	60	48.0	51.0	9.10 × 10^3^	1.38	[63]
45		MMA	FeBr_3_(HIDipp)	TBABr	EBPA	0.2	60	24.0	64.0	1.29 × 10^4^	1.20	[65]
46		MMA	FeCl_3_	HMTA	CCl_4_	N/A	60	5.0	71.1	3.55 × 10^4^	1.25	[102]
47		styrene	FeCl_3_	TDA	PEBr	14.5	110	96.0	26.2	7.85 × 10^3^	1.12	[60]
48		styrene	FeBr_3_	TBABr	EBPA	0.4	90	24.0	70.0	1.40 × 10^4^	1.15	[64]
49	AGET ATRP	MMA	FeCl_3_	IDA	EBiB	28.0	90	7.0	76.0	2.50 × 10^4^	1.30	[68]
50		MMA	FeCl_3_	TBABr	EBiB	18.8	90	7.0	55.8	3.45 × 10^4^	1.21	[69]
51		MMA	FeCl_3_	TPP	EBiB	18.8	90	14.0	44.3	2.18 × 10^4^	1.25	[70]
52		MMA	FeBr_3_	DMF	EBPA	11.8	60	10.0	35.3	3.14 × 10^4^	1.23	[129]
53		MMA	FeBr_3_	TnBP	EBiB	23.4	80	2.5	84.0	2.02 × 10^4^	1.23	[74]
54		MMA	FeBr_3_	TMP	EBiB	23.4	80	2.0	54.0	1.11 × 10^4^	1.26	[74]
55		MMA	FeBr_3_	DPPP	EBiB	23.4	80	9.0	87.0	1.75 × 10^4^	1.18	[74]
56		MMA	FeBr_3_	BDPPM	EBiB	23.4	80	1.0	71.0	7.30 × 10^3^	1.23	[74]
57		MMA	FeBr_3_	BDPPE	EBiB	23.4	80	4.0	66.0	6.80 × 10^3^	1.49	[74]
58		MMA	FeBr_3_	BDPPP	EBiB	23.4	80	4.0	92.0	9.40 × 10^3^	1.66	[74]
59		MMA	FeCl_3_	TBABr	EBiB	37.7	60	7.0	27.4	9.11 × 10^3^	1.49	[76]
60		MMA	FeCl_3_	[Bmim][CO_3_]	EBiB	37.7	70	18.0	16.7	6.33 × 10^3^	1.33	[77]
61		MMA	FeCl_3_	[Bmim][PO_4_]	EBiB	37.7	90	18.0	43.1	1.12 × 10^4^	1.42	[77]
62		MMA	FeCl_3_	[Bmim][HCO_3_]	EBiB	37.7	90	18.0	31.9	9.24 × 10^3^	1.44	[77]
63	AGET ATRP	MMA	FeCl_3_	BMIMPF_6_	EBiB	12.6	90	4.0	46.2	2.31 × 10^4^	1.23	[79]
64		styrene	FeCl_3_	TDA	BMPB	13.1	110	8.6	65.4	1.49 × 10^4^	1.15	[72]
65		styrene	FeBr_3_	TnBA	BEB ^d^	21.8	110	2.0	68.0	2.67 × 10^4^	1.20	[73]
66		styrene	FeCl_3_	TBABr	PEBr	17.5	110	24.0	46.7	9.10 × 10^3^	1.28	[75]
67		tBS	FeBr_3_	TnBA	BEB ^d^	54.6	110	2.0	51.0	1.19 × 10^4^	1.33	[73]
68		MS	FeBr_3_	TnBA	BEB ^d^	38.0	110	2.0	61.0	1.28 × 10^4^	1.38	[73]
69		AS	FeBr_3_	TnBA	BEB ^d^	58.2	110	2.0	85.0	1.36 × 10^4^	1.32	[73]
70	GAMA ATRP	MMA	FeBr_3_	DPPP	EBiB	4.7	80	4.0	42.0	5.10 × 10^3^	1.16	[80]
71		MMA	FeBr_3_	DPPP	EBiB	23.4	80	6.0	57.0	1.24 × 10^4^	1.15	[81]
72		MMA	FeCl_3_	TPP	EBiB	47.1	80	5.0	62.0	2.62 × 10^4^	1.13	[82]
73		MMA	FeCl_3_	TnBA	(MMA)_2_-Cl ^e^	10.0	100	76.0	91.0	9.70 × 10^3^	1.26	[85]
74		MMA	FeBr_3_	TPP	/	47.1	80	0.5	11.0	6.01 × 10^4^	1.21	[87]
75		styrene	FeBr_3_	DPPP	PEBr	87.0	110	12.0	54.0	6.75 × 10^3^	1.12	[80]
76		styrene	FeCl_3_	DPPDMA	PECl	87.3	110	15.0	39.0	4.61 × 10^3^	1.10	[83]
77		styrene	FeCl_3_	TnBP	(MMA)_2_-Cl ^e^	10.0	100	N/A	91.0	1.10 × 10^4^	1.19	[84]
78		styrene	FeBr_3_	TTMPP	EBiB	21.8	100	21.0	44.0	8.10 × 10^3^	1.11	[86]
79		MA	FeBr_3_	DPPP	EBiB	55.5	80	24.0	40.0	4.30 × 10^3^	1.18	[80]
80	SARA ATRP	MMA	FeBr_3_/Fe^0^	TBABr	EBPA	0.6	60	45.0	76.0	1.64 × 10^4^	1.18	[63]
81		MA	CuBr_2_/Fe^0^	Me_6_TREN	MBP	0.7	25	72.0	88.0	1.78 × 10^4^	1.06	[88]
82		MA	CuBr_2_/Fe^0^	Me_6_TREN	EBiB	N/A	30	5.0	77.0	1.54 × 10^4^	1.08	[90]
83		DMAEMA	CuBr_2_/Fe^0^	PMDETA	EBiB	4.4	25	N/A	93.0	1.40 × 10^4^	1.13	[89]

^a^ CCl_4_ = carbon tetrachloride; ^b^ (MMA)_2_Br = Me_2_C(CO_2_Me)CH_2_C-(CO_2_Me)(Me)Br; ^c^ H-(MMA)_2_-Br = H(CH_2_CMeCO_2_Me)_2_Br; ^d^ BEB = 1-bromoethyl benzene; ^e^ (MMA)_2_-Cl = Me_2_C(CO_2_Me)CH_2_C-(CO_2_Me)(Me)Cl.

**Table 2:** Enzyme catalyzed ATRP.

Entry	Monomer	Catalyst	Reducing Agent/Additive	Initiator	Catalyst Concentration	Temp. (°C)	Time (h)	Conv. (%)	*M*_n_ (g/mol)	*M*_w_/*M*_n_	Ref.
1	PEGMA	LTV	VC	BPN	4.00 mg/mL	40	0.5	28.0	1.71 × 10^5^	1.94	[131]
2	PEGMA	LTV	VC	EBiB	4.00 mg/mL	40	1.0	20.0	2.72 × 10^5^	2.43	[131]
3	PEGMA	LTV	VC	EIAc	4.00 mg/mL	40	22.0	15.0	4.32 × 10^5^	2.27	[131]
4	PEGMA	Hb	VC	HEBiB	2.50 mg/mL	25	4.0	48.3	5.00 × 10^3^	1.14	[134]
5	PEGMA	DhHP-6	sodium L-ascorbate	EBiB	1.40 mg/mL	35	2.0	80.7	6.02 × 10^3^	1.08	[138]
6	PEGMA	DhHP-6@ZIF-8	L-ascorbate	BPN	N/A	30	4.0	85.5	8.20 × 10^3^	1.10	[139]
7	PEGA	CBL	VC	BPN	8.00 mg/mL	40	8.0	81.0	1.18 × 10^4^	1.66	[132]
8	PEGA	CBL	VC	EBiB	8.00 mg/mL	40	8.0	50.0	9.81 × 10^3^	1.61	[132]
9	PEGA	LTV	VC	BPN	4.00 mg/mL	40	N/A	76.0	1.10 × 10^4^	1.63	[132]
10	PEGA	HRP	VC	BPN	0.80 mg/mL	40	N/A	62.0	9.63 × 10^3^	1.58	[132]
11	PEGA	Hb	VC	HEBiB	2.50 mg/mL	25	6.0	56.0	6.60 × 10^3^	1.41	[134]
12	NIPAAm	HRP	L-ascorbate	HEBiB	N/A	25	24.0	48.0	9.99 × 10^4^	1.44	[133]
13	NIPAAm	Hb	VC	HEBiB	2.95 mg/mL	25	4.0	60.1	2.93 × 10^5^	1.73	[134]
14	NIPAAm	hematin	sodium L-ascorbate	EBiB	2.53 mg/mL	25	24.0	80.0	3.18 × 10^4^	1.80	[136]
15	OEOMA	mesohemin-(MPEG_550_)_2_	sodium L-ascorbate	PEG_2000_-Br ^a^	2.00 mmol/L	30	6.0	60.0	6.30 × 10^4^	1.19	[137]
16	OEOMA	hemin	sodium L-ascorbate	PEG_2000_-Br ^a^	2.00 mmol/L	30	18.0	50.0	6.00 × 10^4^	1.32	[137]
17	OEOMA	HRP	acetylacetonate	EBPA	270.00 nmol/L	37	0.5	58.0	3.84 × 10^4^	1.13	[142]
18	BMA	GOx	sodium pyruvate	EBPA	2.00 μmol/L	44	6.5	89.0	3.24 × 10^4^	1.16	[141]
19	NIPAAm	Hb	sodium nitrate	BIBB	N/A	25	16.7	N/A	N/A	N/A	[147]

^a^ PEG_2000_ = Polyethylene glycol 2000.

**Table 3:** Metal-free catalyst mediated ATRP.

Entry	Monomer	Catalyst	Light Source	Initiator	Catalyst Concentration (mmol/L)	Temp. (°C)	Time (h)	Conv. (%)	*M*_n_ (g/mol)	*M*_w_/*M*_n_	Ref.
1	BnMA	Ph-PTZ	UV light	EBPA	5.9	25	9.0	70.1	1.40 × 10^4^	1.36	[149]
2	AN	Ph-PTZ	UV light	EBPA	5.0	N/A	7.0	63.0	1.21 × 10^4^	1.42	[150]
3	AN	4-MeOPh-PTZ	UV light	EBPA	5.0	N/A	6.0	33.0	7.49 × 10^3^	1.69	[150]
4	AN	Nap-PTZ	UV light	EBPA	5.0	N/A	15.0	42.0	8.14 × 10^3^	1.62	[150]
5	MMA	Ph-PTZ	UV light	EBPA	4.7	25	4.0	16.0	2.07 × 10^3^	1.50	[151]
6	MMA	Ph-PTZ	UV light	EBiB	4.7	25	4.0	20.0	3.84 × 10^3^	1.79	[151]
7	MMA	Nap-PTZ	UV light	EBPA	4.7	25	4.0	10.0	1.60 × 10^3^	1.40	[151]
8	MMA	PDPA	UV light	PhBMP	3.1	25	24.0	94.6	1.17 × 10^4^	1.46	[152]
9	MMA	perylene	natural sunlight	EBPA	5.4	N/A	10.0	59.2	4.12 × 10^4^	1.29	[153]
10	MMA	anthracene	UV light	EBP	47.1	N/A	2.0	10.1	8.70 × 10^3^	1.41	[154]
11	MMA	erythrosin B	visible light	EBP	3.1	25	2.0	20.0	9.00 × 10^4^	1.20	[156]
12	MMA	phenN-CF_3_	white LED	EBPA	9.4	N/A	8.0	98.4	1.53 × 10^4^	1.17	[157]
13	MMA	4CzIPN	blue LED	EBPA	0.1	25	3.0	90.0	1.91 × 10^4^	1.50	[159]
14	MMA	p-anisaldehyde	CFL bulbs	CF_3_(CF_2_)_5_-I	N/A	N/A	46.0	77.9	3.04 × 10^4^	1.47	[160]
15	MMA	TBOC-QA	blue LED	EBPA	3.8	N/A	10.0	66.3	1.54 × 10^4^	1.56	[161]
16	DMAEMA	PDPA	UV light	PhBMP	3.1	25	24.0	80.2	1.35 × 10^5^	1.36	[152]
17	HEMA	PDPA	UV light	PhBMP	3.1	25	24.0	51.0	7.05 × 10^3^	1.48	[152]
18	DEAEMA	Ph-PTZ	380 nm LED light	EBiB	3.8	25	6.0	48.2	3.98 × 10^3^	1.47	[166]
19	GMA	Ph-PTZ	380 nm LED light	EBiB	3.8	25	6.0	17.4	2.14 × 10^3^	1.60	[166]
20	AMA	Ph-PTZ	380 nm LED light	EBiB	3.8	25	6.0	11.1	2.01 × 10^3^	1.50	[166]
